# Crystal structure of 4-methyl­sulfanyl-2-phenyl­quinazoline

**DOI:** 10.1107/S1600536814015657

**Published:** 2014-07-23

**Authors:** Mohammed B. Alshammari, Keith Smith, Amany S. Hegazy, Benson M. Kariuki, Gamal A. El-Hiti

**Affiliations:** a Chemistry Department, College of Sciences and Humanities, Salman bin Abdulaziz University, PO Box 83, Al-Kharij 11942, Saudi Arabia; b School of Chemistry, Cardiff University, Main Building, Park Place, Cardiff CF10 3AT, UK; c Cornea Research Chair, Department of Optometry, College of Applied Medical Sciences, King Saud University, PO Box 10219, Riyadh 11433, Saudi Arabia

**Keywords:** crystal structure, methylthioquinazoline, π–π stacking

## Abstract

In the title compound, C_15_H_12_N_2_S, the methylthioquinazoline group is planar with the methyl C displaced by only 0.116 (3) Å from the plane of the quinazoline moiety. The dihedral angle between the phenyl ring and the quinazoline ring system is 13.95 (5)°. In the crystal, each molecule is linked by π–π stacking between to two adjacent inversion-related molecules. On one side, the inverted quinazoline groups interact with a centroid–centroid distance of 3.7105 (9) Å. On the other side, the quinazoline group interacts with the pyrimidine and phenyl rings of the second neighbour with centroid–centroid distances of 3.5287 (8) and 3.8601 (9) Å, respectively.

## Related literature   

For the synthesis of 4-alkythio­qinazolines, see: Leonard & Curtin (1946 ▶); Hearn *et al.* (1951 ▶); Meerwein *et al.* (1956 ▶); Blatter & Lukaszewski (1964 ▶); Segarra *et al.* (1998 ▶); Smith *et al.* (2005*a* ▶,*b* ▶).
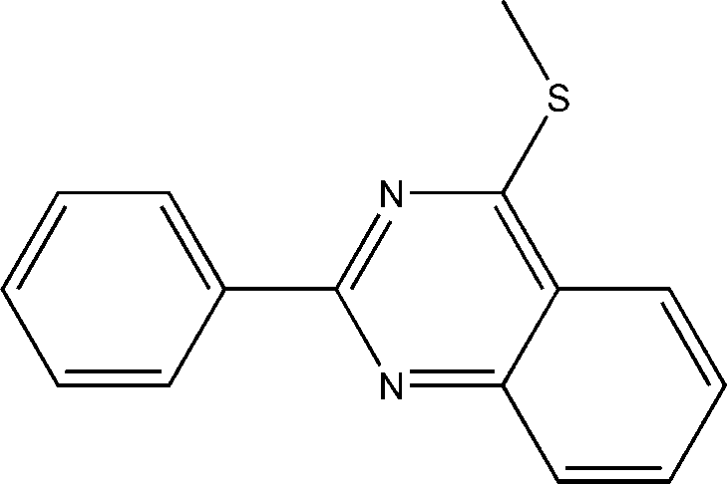


## Experimental   

### Crystal data  

C_15_H_12_N_2_S
*M* *_r_* = 252.33
Monoclinic, 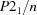

*a* = 10.1951 (3) Å
*b* = 7.3545 (2) Å
*c* = 16.5300 (5) Å
β = 102.860 (3)°
*V* = 1208.33 (6) Å^3^
*Z* = 4
Mo *K*α radiation
μ = 0.25 mm^−1^
*T* = 150 K
0.23 × 0.18 × 0.15 mm

### Data collection  

Agilent SuperNova (Dual, Cu at zero, Atlas) diffractometer
Absorption correction: multi-scan (*CrysAlis PRO*; Agilent, 2013 ▶) *T*_min_ = 0.848, *T*_max_ = 1.000
11140 measured reflections
3025 independent reflections
2558 reflections with *I* > 2σ(*I*)
*R*_int_ = 0.030

### Refinement  

*R*[*F* ^2^ > 2σ(*F* ^2^)] = 0.039
*wR*(*F* ^2^) = 0.097
*S* = 1.08
3025 reflections
164 parameters
H-atom parameters constrained
Δρ_max_ = 0.29 e Å^−3^
Δρ_min_ = −0.34 e Å^−3^

### 

Data collection: *CrysAlis PRO* (Agilent, 2013 ▶); cell refinement: *CrysAlis PRO*; data reduction: *CrysAlis PRO*; program(s) used to solve structure: *SHELXTL* (Sheldrick, 2008 ▶); program(s) used to refine structure: *SHELXTL*; molecular graphics: *SHELXTL*; software used to prepare material for publication: *SHELXTL*.

## Supplementary Material

Crystal structure: contains datablock(s) I, New_Global_Publ_Block. DOI: 10.1107/S1600536814015657/xu5801sup1.cif

Structure factors: contains datablock(s) I. DOI: 10.1107/S1600536814015657/xu5801Isup2.hkl

Supporting information file. DOI: 10.1107/S1600536814015657/xu5801Isup3.cml

CCDC reference: 1012164

Additional supporting information:  crystallographic information; 3D view; checkCIF report

## Figures and Tables

**Fig. 1.**
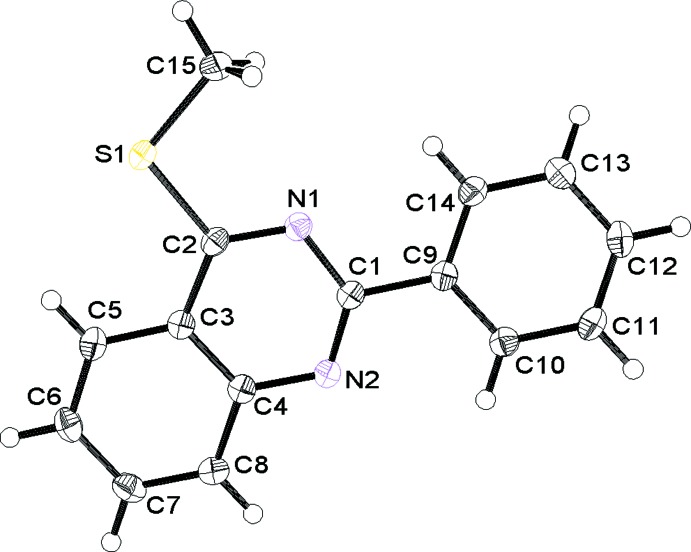
A molecule showing atom labels and 50% probability displacement ellipsoids for non-H atoms.

**Fig. 2.**
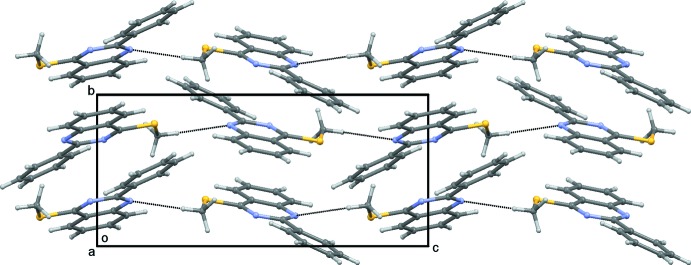
Packing diagram.

## supplementary crystallographic information

### S2. Structural commentary

In the 4-(methyl­thio)-2-phenyl­quinazoline molecule (Fig 1), the angle between the planes through the phenyl and phenyl­quinazoline ring systems is 13.95 (5)°. The molecules are stacked in the [010] direction with approximately parallel molecular planes. With no strong H-bond donors, one N atom accepts a long C—H···N contact linking molecules along [101]. The second N atom is not involved. 4-Methyl­thio­quinazoline derivatives can be obtained from reaction of the potassium salt of 3*H*-quinazoline-4-thio­nes with iodo­methane (Leonard & Curtin, 1946; Meerwein *et al.*, 1956). Quinazoline-4-thio­nes are produced from the corresponding 3*H*-quinazoline-4-ones using phospho­rus penta­sulfide (Hearn, *et al.*, 1951), Lawesson's reagent (Segarra *et al.*, 1998) or iso­thio­cyanates (Blatter & Lukaszewski, 1964). In a continuation of our research focused on new synthetic routes towards novel substituted 4-alkyl­thio­quinazoline derivatives (Smith *et al.*, 2005**a*,b*) we have synthesized 4-(methyl­thio)-2-phenyl­quinazoline in a high yield (Smith *et al.*, 2005*a*).

### S5. Synthesis and crystallization

To a solution of 2-phenyl-3*H*-quinazoline-4-thione (4.81 g, 20.2 mmol) in a 1:1 mixture of MeOH and water (50 ml) containing KOH (3.0 g), was added iodo­methane (3.41 g, 24.0 mmol). The reaction mixture was stirred for 20 min at room temperature and the solid obtained was filtered, washed with H_2_O (3 × 30 ml), dried and recrystallized from Et_2_O to give 4-(methyl­thio)-2-phenyl­quinazoline (4.63 g, 18.3 mmol, 91%) as colourless crystals, m.p. 93-94 °C [lit. 94 °C (H_2_O); Meerwein *et al.*, 1956). ^1^H NMR (400 MHz, CDCl_3_, δ, p.p.m.) 8.70-8.66 (m, 2 H, ArH), 8.10-8.03 (m, 2 H, ArH), 7.83 (app. dt, *J* = 1, 8 Hz, 1 H, H-7), 7.58-7.51 (m, 4 H, ArH), 2.85 (s, 3 H, CH_3_). ^13^C NMR (100 MHz, CDCl_3_, *d*, p.p.m.) 171.8 (s, C-2), 159.2 (s, C-4), 149.1 (s, C-8a), 138.5 (s, C-1 of Ph), 133.9 (d, C-7), 131.0 (d, C-4 of Ph), 129.4 (d, C-8), 129.0 (d, C-3/C-5 of Ph), 128.9 (d, C-2/C-6 of Ph), 127.1 (d, C-6), 124.1 (d, C-5), 123.0 (s, C-4a), 13.0 (q, CH_3_). EI—MS (*m/z*, %): 252 (*M*^+^, 100), 251 (72), 205 (60), 102 (47), 77 (61), 51 (33). CI—MS (*m/z*, %): 253 (*MH*^+^, 100), 207 (3). HRMS (CI): Calculated for C_15_H_13_N_2_S [*MH*] 253.0794; found, 253.0789.

### S6. Refinement

H atoms were placed in calculated positions with C—H = 0.95 and 0.98 Å and refined in riding mode, U_iso_(H) = 1.5U_eq_(C) for methyl H atoms and 1.2U_eq_(C) for aromatic H atoms.

### Crystal data

**Table:**

C_15_H_12_N_2_S	*F*(000) = 528
*M**_r_* = 252.33	*D*_x_ = 1.387 Mg m^−^^3^
Monoclinic, *P*2_1_/*n*	Mo *K*α radiation, λ = 0.71073 Å
*a* = 10.1951 (3) Å	Cell parameters from 2558 reflections
*b* = 7.3545 (2) Å	θ = 3.1–29.7°
*c* = 16.5300 (5) Å	µ = 0.25 mm^−^^1^
β = 102.860 (3)°	*T* = 150 K
*V* = 1208.33 (6) Å^3^	Block, colourless
*Z* = 4	0.23 × 0.18 × 0.15 mm

### Data collection

**Table:**

Agilent SuperNova (Dual, Cu at zero, Atlas) diffractometer	3025 independent reflections
Radiation source: SuperNova (Mo) X-ray Source	2558 reflections with *I* > 2σ(*I*)
Mirror monochromator	*R*_int_ = 0.030
ω scans	θ_max_ = 29.7°, θ_min_ = 3.1°
Absorption correction: multi-scan (*CrysAlis PRO*; Agilent, 2013)	*h* = −13→12
*T*_min_ = 0.848, *T*_max_ = 1.000	*k* = −9→10
11140 measured reflections	*l* = −17→22

### Refinement

**Table:**

Refinement on *F*^2^	0 restraints
Least-squares matrix: full	Hydrogen site location: inferred from neighbouring sites
*R*[*F*^2^ > 2σ(*F*^2^)] = 0.039	H-atom parameters constrained
*wR*(*F*^2^) = 0.097	*w* = 1/[σ^2^(*F*_o_^2^) + (0.0323*P*)^2^ + 0.6772*P*] where *P* = (*F*_o_^2^ + 2*F*_c_^2^)/3
*S* = 1.08	(Δ/σ)_max_ < 0.001
3025 reflections	Δρ_max_ = 0.29 e Å^−^^3^
164 parameters	Δρ_min_ = −0.34 e Å^−^^3^

### Special details

**Table:**

Geometry. All e.s.d.'s (except the e.s.d. in the dihedral angle between two l.s. planes) are estimated using the full covariance matrix. The cell e.s.d.'s are taken into account individually in the estimation of e.s.d.'s in distances, angles and torsion angles; correlations between e.s.d.'s in cell parameters are only used when they are defined by crystal symmetry. An approximate (isotropic) treatment of cell e.s.d.'s is used for estimating e.s.d.'s involving l.s. planes.

### Fractional atomic coordinates and isotropic or equivalent isotropic displacement parameters (Å^2^)

**Table:**

	*x*	*y*	*z*	*U*_iso_*/*U*_eq_	
C1	0.09137 (15)	0.6738 (2)	−0.06125 (9)	0.0182 (3)	
C2	0.06916 (15)	0.7676 (2)	0.06700 (9)	0.0189 (3)	
C3	−0.07074 (14)	0.8041 (2)	0.03446 (9)	0.0183 (3)	
C4	−0.11800 (15)	0.7663 (2)	−0.05066 (9)	0.0196 (3)	
C5	−0.16032 (16)	0.8695 (2)	0.08196 (10)	0.0229 (3)	
H5	−0.1285	0.8950	0.1393	0.027*	
C6	−0.29284 (16)	0.8957 (2)	0.04503 (10)	0.0257 (3)	
H6	−0.3531	0.9391	0.0770	0.031*	
C7	−0.34049 (16)	0.8591 (2)	−0.03973 (11)	0.0266 (4)	
H7	−0.4328	0.8783	−0.0646	0.032*	
C8	−0.25564 (15)	0.7960 (2)	−0.08710 (10)	0.0246 (3)	
H8	−0.2892	0.7723	−0.1445	0.030*	
C9	0.18424 (15)	0.5989 (2)	−0.11089 (9)	0.0188 (3)	
C10	0.13360 (15)	0.5219 (2)	−0.18889 (9)	0.0213 (3)	
H10	0.0394	0.5222	−0.2116	0.026*	
C11	0.22001 (16)	0.4449 (2)	−0.23338 (9)	0.0237 (3)	
H11	0.1846	0.3913	−0.2860	0.028*	
C12	0.35767 (16)	0.4460 (2)	−0.20139 (10)	0.0253 (3)	
H12	0.4166	0.3939	−0.2321	0.030*	
C13	0.40914 (16)	0.5234 (2)	−0.12434 (10)	0.0250 (3)	
H13	0.5036	0.5249	−0.1025	0.030*	
C14	0.32309 (15)	0.5986 (2)	−0.07900 (9)	0.0225 (3)	
H14	0.3589	0.6502	−0.0260	0.027*	
C15	0.30675 (16)	0.7320 (3)	0.18247 (10)	0.0289 (4)	
H15A	0.3070	0.6036	0.1666	0.043*	
H15B	0.3564	0.7464	0.2401	0.043*	
H15C	0.3498	0.8048	0.1460	0.043*	
N1	0.14809 (12)	0.70406 (17)	0.02073 (8)	0.0191 (3)	
N2	−0.03494 (12)	0.70111 (17)	−0.09896 (8)	0.0200 (3)	
S1	0.13627 (4)	0.80726 (6)	0.17278 (2)	0.02388 (12)	

### Atomic displacement parameters (Å^2^)

**Table:**

	*U*^11^	*U*^22^	*U*^33^	*U*^12^	*U*^13^	*U*^23^
C1	0.0219 (7)	0.0154 (7)	0.0174 (7)	−0.0014 (5)	0.0049 (6)	0.0015 (6)
C2	0.0237 (7)	0.0172 (7)	0.0156 (7)	−0.0014 (6)	0.0042 (6)	0.0017 (6)
C3	0.0216 (7)	0.0147 (7)	0.0191 (7)	−0.0010 (5)	0.0058 (6)	0.0020 (6)
C4	0.0218 (7)	0.0173 (7)	0.0202 (7)	−0.0011 (6)	0.0055 (6)	0.0014 (6)
C5	0.0282 (8)	0.0212 (8)	0.0208 (8)	0.0013 (6)	0.0088 (6)	0.0005 (6)
C6	0.0254 (8)	0.0229 (8)	0.0316 (9)	0.0035 (6)	0.0126 (7)	−0.0004 (7)
C7	0.0195 (7)	0.0261 (8)	0.0335 (9)	0.0018 (6)	0.0043 (6)	0.0005 (7)
C8	0.0222 (8)	0.0274 (9)	0.0231 (8)	−0.0008 (6)	0.0024 (6)	−0.0016 (7)
C9	0.0232 (7)	0.0161 (7)	0.0176 (7)	0.0008 (6)	0.0059 (6)	0.0026 (6)
C10	0.0229 (7)	0.0216 (8)	0.0195 (7)	−0.0004 (6)	0.0048 (6)	0.0022 (6)
C11	0.0318 (8)	0.0225 (8)	0.0174 (7)	−0.0004 (6)	0.0067 (6)	−0.0011 (6)
C12	0.0300 (8)	0.0234 (8)	0.0256 (8)	0.0037 (6)	0.0131 (7)	0.0006 (7)
C13	0.0226 (8)	0.0258 (9)	0.0274 (8)	0.0019 (6)	0.0071 (6)	0.0017 (7)
C14	0.0252 (8)	0.0230 (8)	0.0188 (7)	0.0001 (6)	0.0039 (6)	−0.0002 (6)
C15	0.0241 (8)	0.0406 (10)	0.0202 (8)	0.0037 (7)	0.0011 (6)	0.0014 (7)
N1	0.0209 (6)	0.0198 (6)	0.0171 (6)	−0.0007 (5)	0.0050 (5)	0.0019 (5)
N2	0.0218 (6)	0.0200 (6)	0.0185 (6)	−0.0005 (5)	0.0051 (5)	−0.0009 (5)
S1	0.0248 (2)	0.0308 (2)	0.0159 (2)	0.00122 (15)	0.00423 (15)	−0.00238 (16)

### Geometric parameters (Å, º)

**Table:**

C1—N2	1.3153 (19)	C8—H8	0.9500
C1—N1	1.3680 (19)	C9—C14	1.396 (2)
C1—C9	1.4897 (19)	C9—C10	1.398 (2)
C2—N1	1.3134 (18)	C10—C11	1.388 (2)
C2—C3	1.433 (2)	C10—H10	0.9500
C2—S1	1.7544 (15)	C11—C12	1.385 (2)
C3—C4	1.410 (2)	C11—H11	0.9500
C3—C5	1.414 (2)	C12—C13	1.387 (2)
C4—N2	1.3730 (18)	C12—H12	0.9500
C4—C8	1.415 (2)	C13—C14	1.389 (2)
C5—C6	1.367 (2)	C13—H13	0.9500
C5—H5	0.9500	C14—H14	0.9500
C6—C7	1.403 (2)	C15—S1	1.7970 (16)
C6—H6	0.9500	C15—H15A	0.9800
C7—C8	1.370 (2)	C15—H15B	0.9800
C7—H7	0.9500	C15—H15C	0.9800
			
N2—C1—N1	126.73 (13)	C10—C9—C1	120.56 (13)
N2—C1—C9	118.08 (13)	C11—C10—C9	120.43 (14)
N1—C1—C9	115.18 (13)	C11—C10—H10	119.8
N1—C2—C3	122.39 (13)	C9—C10—H10	119.8
N1—C2—S1	119.07 (11)	C12—C11—C10	120.27 (15)
C3—C2—S1	118.54 (11)	C12—C11—H11	119.9
C4—C3—C5	120.11 (14)	C10—C11—H11	119.9
C4—C3—C2	115.34 (13)	C11—C12—C13	119.80 (14)
C5—C3—C2	124.53 (14)	C11—C12—H12	120.1
N2—C4—C3	122.09 (13)	C13—C12—H12	120.1
N2—C4—C8	119.20 (14)	C12—C13—C14	120.23 (15)
C3—C4—C8	118.71 (13)	C12—C13—H13	119.9
C6—C5—C3	119.75 (15)	C14—C13—H13	119.9
C6—C5—H5	120.1	C13—C14—C9	120.38 (14)
C3—C5—H5	120.1	C13—C14—H14	119.8
C5—C6—C7	120.47 (14)	C9—C14—H14	119.8
C5—C6—H6	119.8	S1—C15—H15A	109.5
C7—C6—H6	119.8	S1—C15—H15B	109.5
C8—C7—C6	120.88 (15)	H15A—C15—H15B	109.5
C8—C7—H7	119.6	S1—C15—H15C	109.5
C6—C7—H7	119.6	H15A—C15—H15C	109.5
C7—C8—C4	120.08 (15)	H15B—C15—H15C	109.5
C7—C8—H8	120.0	C2—N1—C1	117.13 (13)
C4—C8—H8	120.0	C1—N2—C4	116.31 (13)
C14—C9—C10	118.88 (13)	C2—S1—C15	101.10 (7)
C14—C9—C1	120.52 (13)		

### Hydrogen-bond geometry (Å, º)

**Table:**

*D*—H···*A*	*D*—H	H···*A*	*D*···*A*	*D*—H···*A*
C15—H15*B*···N2^i^	0.98	2.67	3.648 (2)	173

Symmetry code: (i) *x*+1/2, −*y*+3/2, *z*+1/2.
